# The Examination of Aviators

**Published:** 1918

**Authors:** 


***The Examination of Aviators.*** By F. A. Bachman. Abs. from the *U. S. Naval Medical Bulletin,* Jan. 1918.

The writer considers it important to test the emotional apparatus of the flier as well as his physical fitness.

A new departure in physical examinations is the introduction of the Barany test of the vestibular function[1].

The procedure of examination is somewhat as follows.

*General examination* : Height, weight, chest, lungs, bones, joints, skin, nervous system, vertigo, vascular system, digestive system, urinalysis, Wassermanns.

*Dynamic tests* : Walking in a straight line forward and backward for twenty feet, maintaining a position for one minute without swaying.

*Vision tests* : Stereoscopic vision should be perfect. Ocular movements may be tested by having the eyes follow the movement of the tester's finger in all directions. The movements should be regular and identical. Pupillary reactions should be regular and equal in each eye when responding to direct or indirect light stimulation and to accommodation. Intraocular tension may be tested roughly by palpating the eye with the index finger of each hand through the upper lid, as the candidate looks downward. The tension of one eye should be compared with that of the other and with that of an eye believed to be normal. If the tension is not normal it is a cause for rejection. Ocular nystagmus if rythmical and if it occurs upon looking straight ahead or laterally ($40^0$ or less) is a cause for rejection. A restricted field of vision in either eye, if confirmed by a perimeter, constitutes cause for rejection. The Jennings test cards are used for color perception. The candidate punches through the perforation and so makes a permanent record card.

Muscular balance, at 20 feet. Hyperphoria, exophoria, esophoria. Muscular imbalance may be determined by a phorometer.

Visual acuity. 25/20 required for each eye (Snellen's type 20 feet). The near point should not be more than 11 cm., at 20 yrs., 13 cm. at 25 yrs., and 15 cm. at 30 yrs.

Ophthalmoscopic findings. One drop of a 5 per cent. solution

1. See p. 563.

of euphthalmine in each eye held with the eye closed for fifteen minutes and then another drop in each eye held for fifteen minutes more, may cause a pathological condition of the fundus : if so, it is cause for rejection.

*Ear tests* : The history of ear troubles should be noted, such as, discharge, buzzing, ringing, noise, vertigo, seasickness, cranial injury. The appearance of the ex-auditory canal and of the membrani tympani should also be noted. The hearing for each ear should be normal, and can be tested by the whisper and watch methods, although the writer considers the watch method the more reliable. The tick of an Ingersoll watch ought to be heard at a distance of about 40 inches.

The Eustachian tubes should be examined per catheter, and also there should be an examination for diseased tonsils, adnoids, etc.

*Equilibrium.* The Barany chair method is used for this test[1]. Nystagmus, pointing, falling.

*Psychological tests.* These are carried out by means of a Hipp chronoscope or a d'Arsonval chronometer. The author expresses some doubt as to the value of this test for the psychological reactions. He thinks it would be more valuable if some sort of test were devised to determine the emotional characteristics of the candidate and also the accuracy of his judgment. These are qualifications which the instructor is quick to observe. At the Naval Hospital in Philadelphia, an attempt has recently been made to measure the attribute of judgment. Candidates have been asked to estimate the length of sticks of various size when compared with a yard stick. Their sense of time has been tested by having them estimate the time required for sand to pass through a time glass. Speed judgment was tested by displaying a series of three revolving 9-inch discs, rotated at various but slightly different rates of speed. The candidate was required to arrange the disks in the order of their speed. Judgment of arrangement was tested by a square of wood sawed into seven pieces. The time necessary for the candidate to arrange the pieces so as to make a perfect square was noted. The results obtained by testing one hundred men in this fashion, the author believes, warrants the institution of such tests in determining fitness in aviation.

1. See p. 563.

